# Factors and HCV treatment outcomes associated with smoking among people who inject drugs on opioid agonist treatment: secondary analysis of the PREVAIL randomized clinical trial

**DOI:** 10.1186/s12879-020-05667-3

**Published:** 2020-12-04

**Authors:** Irene Pericot-Valverde, Moonseong Heo, Matthew J. Akiyama, Brianna L. Norton, Linda Agyemang, Jiajing Niu, Alain H. Litwin

**Affiliations:** 1 School of Health Research, Clemson University, Clemson, SC USA; 2 Department of Medicine, Prisma Health, Greenville, SC USA; 3 Department of Public Health Sciences, College of Behavioral, Social, and Health Sciences, Clemson University, Clemson, SC USA; 4 Albert Einstein College of Medicine, Montefiore Medical Center, Bronx, NY USA; 5 School of Mathematical and Statistical Sciences, College of Science, Clemson University, Clemson, SC USA; 6 Department of Medicine, University of South Carolina School of Medicine, Greenville, SC USA

**Keywords:** HCV, DAA, Smoking, SVR, PWID

## Abstract

**Background:**

Cigarette smoking has emerged as a leading cause of mortality among people with hepatitis C virus (HCV). People who inject drugs (PWID) represent the largest group of adults infected with HCV in the US. However, cigarette smoking remains virtually unexplored among this population. This study aimed at (1) determining prevalence and correlates of cigarette smoking among HCV-infected PWID enrolled in opiate agonist treatment programs; (2) exploring the association of smoking with HCV treatment outcomes including adherence, treatment completion and sustained virologic response (SVR); and 3) exploring whether cigarette smoking decreased after HCV treatment.

**Methods:**

Participants were 150 HCV-infected PWID enrolled in a randomized clinical trial primarily designed to test three intensive models of HCV care. Assessments included sociodemographics, presence of chronic health and psychiatric comorbidities, prior and current drug use, quality of life, and HCV treatment outcomes.

**Results:**

The majority of the patients (84%) were current cigarette smokers at baseline. There was a high prevalence of psychiatric and medical comorbidities in the overall sample of PWID. Alcohol and cocaine use were identified as correlates of cigarette smoking. Smoking status did not influence HCV treatment outcomes including adherence, treatment completion and SVR. HCV treatment was not associated with decreased cigarette smoking.

**Conclusions:**

The present study showed high prevalence of cigarette smoking among this population as well as identified correlates of smoking, namely alcohol and cocaine use. Cigarette smoking was not associated with HCV treatment outcomes. Given the detrimental effects that cigarette smoking and other co-occurring, substance use behaviors have on HCV-infected individuals’ health, it is imperative that clinicians treating HCV also target smoking, especially among PWID. The high prevalence of cigarette smoking among PWID will contribute to growing morbidity and mortality among this population even if cured of HCV. Tailored smoking cessation interventions for PWID along with HCV treatment may need to be put into clinical practice.

**Trial registration:**

NCT01857245. Registered May 20, 2013.

## Background

Hepatitis C virus (HCV) infection is a serious, chronic health condition that currently affects approximately 2.5–4.7 million people in the United States [1]. Despite the availability of highly effective oral direct-acting antiviral (DAA) medications with cure rates >95% [2], mortality rates in patients with HCV remain very high and surpasses any other chronic infectious diseases, including human immunodeficiency virus (HIV) [3]. Recently, cigarette smoking has emerged as a leading cause of mortality among people with HCV [4]. Epidemiological data suggests that many chronic health conditions associated with cigarette smoking, such as non-hepatic cancers, cardiovascular disease, and respiratory conditions, have become a major cause of mortality among the HCV-infected population [5, 6]. In addition, cigarette smoking is associated with life-threatening complications among individuals with HCV, including liver fibrosis, cirrhosis, and hepatocellular carcinoma [7–9]. Furthermore, smoking has been associated with lower rates of HCV treatment initiation among patients with chronic HCV [10] and lower rates of sustained viral response (SVR) (i.e., higher treatment failure) to interferon-based HCV treatment among methadone-maintained individuals [11]. Thus, a critical component to improving health and treatment outcomes among people with HCV is to address the disproportionate burden of cigarette smoking in this population compared to the general population.

Limited studies to date have assessed rates of smoking among people infected with HCV in US. For example, only two recent epidemiological studies have estimated the prevalence of cigarette smoking among people with HCV. These studies found that the prevalence of cigarette smoking among individuals with HCV is 3- to 4-fold higher than that of the general US adult population (i.e.,62% vs. 14%, respectively), but did not find differences in the number of cigarettes smoked per day or levels of dependence [12, 13]. However, these prevalence estimates are likely to be underestimated. National datasets do not include HCV-infected marginalized populations, such as people who are incarcerated, homeless, hospitalized, or nursing home residents, which typically present a higher prevalence of smoking, smoke more heavily, and are more dependent [14]. Additionally, very little research has explored correlates of cigarette smoking among people with HCV. These available studies have identified older age, alcohol use, use of illicit substances, and experiencing pain [15, 16] as correlates of cigarette smoking among HCV patients.

Extensive scientific literature has examined the prevalence and correlates of cigarette smoking in individuals with a history of drug injection on opiate agonist treatment (OAT) programs [17–20]. Although the majority of those are infected with HCV, there is limited data focused on exploring the prevalence and factors associated with cigarette smoking among this population, that is, people who inject drugs (PWID) on OAT program. To our knowledge, only one study which explored smoking among people with HCV included some individuals with a history of drug injection [15]. Few studies have examined the association of cigarette smoking and HCV treatment outcomes among PWID, despite the fact that smoking is associated with decreased likelihood of HCV treatment initiation and achievement of virologic response in the HCV-infected population [10, 11], which represents the largest group of adults infected with HCV in the US (50–85%) [21]. We aim at (1) assessing the prevalence and correlates of cigarette smoking among PWID individuals receiving HCV treatment; (2) examining associations between cigarette smoking and HCV treatment outcomes; and 3) exploring whether cigarette smoking decreased after HCV treatment.

## Methods

### Participants

The participants were 150 PWID who enrolled in a randomized clinical trial for HCV treatment (NCT01857245) [22]. This sample size determined based on power analysis was detailed in the related design and rationale paper [23]. The purpose of this trial was to compare the effectiveness of three models of care for HCV treatment: self-administered individual treatment (SIT), group treatment (GT), and directly observed therapy (DOT). Participants were recruited from three opioid treatment programs (OTP) in Bronx, NY. Inclusion criteria for the study were being aged 18 or older, being able to speak English or Spanish, having HCV genotype 1, being psychiatrically stable, willing to receive HCV therapy onsite in their OAT program, being HCV treatment naïve (or treatment experienced with interferon-based regimens), receiving OAT in a OTP clinic, and being able to provide informed consent. Participants were excluded if they had stage B or C liver disease (the most severe form of liver disease) based on Child-Pugh classification for severity of liver disease, were unable to provide informed consent, were currently pregnant or breast-feeding, or were hypersensitive to HCV medication. Participants were randomly assigned in parallel to one of three models of care (DOT, GT, or SIT) on a 1:1:1 allocation ratio basis stratified by site and cirrhosis status.

Research visits were conducted at baseline, every 4 weeks during the first 12 weeks of HCV treatment (treatment week 4, 8, and 12), at the end of treatment (if the treatment regimen was >12 weeks, and 4, 12, and 24 weeks after treatment completion (follow-ups 4, 12, and 24). HCV treatment duration ranged from 8 to 24 weeks (M = 12.9, SD = 5.4) based on the type of regimen indicated for the patient. Detailed information about the study protocol can be found elsewhere [22, 23]. The enrollment period for the clinical trial started on October 2013 and ended on May 2016. Detailed flow chart following the CONSORT guidelines is available in the main outcomes paper [22].

### Data sources

All self-reported measures were collected using pertinent survey instruments employing the Audio Computer-Assisted Self-Interview (ACASI) system. Data about psychiatric and chronic health conditions were obtained through chart review. The medical record of each participant was individually reviewed to determine the presence of any condition. Urine toxicology tests were used to determine recent drug use.

### Outcome measures

#### Smoking-related outcome measures

Participants responded to four questions assessing smoking-related characteristics extracted from the National Health Interview Survey [24]. Two questions assessed history of cigarette smoking (“have you smoked at least 100 cigarettes in your entire life?” and “how old were you when you first started to smoke fairly regularly?” We mean the age when you started smoking cigarettes on a routine basis, not the age when you tried your first cigarette) and the other two on exploring current behavior (“do you now smoke cigarettes every day, some days or not at all?” and “on the average, how many cigarettes do you now smoke a day?”). These questions were administered twice, at baseline and 24 weeks after treatment completion (follow-up 24). Participants who reported smoking at least 100 cigarettes lifetime and now smoking daily or some days were classified as cigarette smokers. Former smokers were defined as those respondents who reported having smoked 100 cigarettes lifetime but not currently smoking. Never cigarette smokers were patients who did not fall into these two categories. For the present study, we combined the former and never smokers with small sample sizes and classified them as non-current smokers.

#### HCV treatment outcome measures

The treatment outcomes included in this study were SVR, treatment completion, and medication adherence. HCV RNA at the post-treatment week 12 was measured using the COBAS TaqMan real-time reverse transcriptase–polymerase chain reaction assay version 1.0 (< 43 IU/mL) or version 2.0 (< 15 IU/mL) after October 2014 (Roche Diagnostics). SVR was declared if HCV ribonucleic acid (RNA) 12 weeks after treatment was undetectable at < 43 IU/mL or < 15 IU/mL. Treatment completion was declared if a participant took at least 80% of the planned duration of treatment. Adherence was defined as the percentage of daily, prescribed doses of the medication actually taken by patients over the planned HCV regimen. Both completion and adherence were measured using electronic Med-ic® blister packs, which have a 99.6% event accuracy [25].

### Potential correlate measures

#### Participants sociodemographics

Participants completed a survey instrument at baseline for sociodemographic characteristics such as age, sex, race/ethnicity, educational attainment, income, and employment status.

#### Drug and alcohol use

We collected information about self-reported drug and alcohol use utilizing the Addiction Severity Index-Lite version (ASI-Lite) [26]. Specifically, we assessed past 30-day use and lifetime use of alcohol, heroin, methadone, opiate, barbiturates, sedatives, cocaine, amphetamines, cannabis, hallucinogens, and inhalants. Additionally, we used urine toxicology screening test (American Biomedica Corporation, Kinderhook, NY) to determine recent use of amphetamines, benzodiazepines, cocaine, and, opiates.

#### Psychiatric and chronic health conditions

Psychiatric conditions included depression, anxiety, psychosis, bipolar disorder, obsessive compulsive disorder (OCD), and post-traumatic stress disorder (PTSD). Chronic health conditions included the following: arthritis, asthma/chronic obstructive pulmonary disease (COPD), congestive heart failure (CHF), diabetes, chronic back pain, chronic pain syndrome, gastroesophageal reflux disease/peptic ulcer disease (GERD/PUD), hypertension, hyperlipidemia, migraine/headaches, sleep apnea, peripheral vascular disease, renal insufficiency, seizure disorder, and thyroid disease.

#### Quality of life

The EQ-5D-3L was used to measure self-reported health-related quality of life in five domains: mobility, self-care, usual activities, pain/discomfort and anxiety/depression [27]. The questionnaire items have three responses for each of the above domains corresponding to three levels of severity: no problems, some/moderate problems, and severe/extreme problems.

#### Covariates

For the comparison of the bi-weekly repeatedly measured adherence rates, we included the following variables as covariates: study arms, alcohol intoxication and psychiatric illness, all of which were shown to be associated with the longitudinal adherence [22].

### Statistical analyses

Descriptive statistics are provided in terms of mean, standard deviation, frequency and percentage in relation to participants’ characteristics at baseline assessment. To identify correlates of baseline current cigarette smoking, chi-square tests for categorical variables and t-test for continuous data were conducted between current and non-smokers for sociodemographics, psychiatric and chronic health conditions, drug and alcohol use related variables, quality of life, and treatment outcomes (overall adherence, treatment completion, and SVR). To compare the bi-weekly measurements of the adherence rates, we applied a mixed-effects linear model with AR(1) covariance structure adjusting for the covariates mentioned above. McNemar’s test was applied to test significance of changes on smoking status between baseline and follow-up week 24. Statistical significance was declared if a two-sided *p*-value is less than .05 and all statistical analyses were conducted using SAS v9.4 (SAS Inc., Cary, NC, USA) R statistical package [28].

## Results

### Sample characteristics

Detailed flow chart following the CONSORT guidelines is available in the main outcomes paper [22]. Table 1 displays descriptive statistics for participants’ sociodemographic characteristics at baseline. Participants were 51.2 (SD = 10.6) years old on average and 64.7% were male. More than half of the sample (56.0%) were Hispanic or Latino and had an annual household income less than $1500. Over three-quarters (81.4%) had a high school diploma or less of education and were unemployed (82.7%).

**Table 1:** Sociodemographic characteristics of the study sample (*N* = 150)

Characteristics	Overall (N = 150) %/M ± SD	Smokers (*n* = 126) n(%)/M ± SD	Non-smokers (*n* = 24) n(%)/M ± SD	*p*
Age	51.2 ± 10.6	50.6 ± 10.5	54.7 ± 10.4	.08
Gender				
Male	97(64.7)	81(64.2)	16(66.6)	.82
Female	53(35.3)	45(35.8)	8(33.4)	
Race/ethnicity				
Black	40(26.7)	35(27.7)	5(20.8)	.30
Hispanic or Latino	84(56.0)	70(55.5)	14(58.3)	
White	12(8.0)	8(6.4)	4(16.7)	
Other	14(9.3)	13(10.4)	1(4.2)	
Marital Status				
Married/living with a partner	55(36.7)	48(38.0)	7(29.1)	.40
Not living with a partner	95(63.3)	78(62.0)	17(70.9)	
Educational attainment				
Less than HS	64(42.7)	55(43.6)	9(37.5)	.64
HS graduate/GED	58(38.7)	49(38.9)	9(37.5)	
≥ HS graduate	28(18.6)	22(17.5)	6(25.0)	
Employment status				
Employed	12(8.0)	8(6.4)	4(16.7)	.12
Unemployed, retired, or disabled	124(82.7)	107(84.9)	17(70.8)	
Retired	14(9.3)	11(8.7)	3(12.5)	
Monthly income				
≤ $1500	89(59.3)	73(57.9)	16(66.7)	.42
≥ $1501	61(40.7)	53(42.1)	8(33.3)	
Income include benefits (e.g., SSI)	67(44.7)	63(50.0)	4(16.7)	.002
Homeless	116(77.3)	96(76.2)	20(83.3)	.44

Note. *%* percentage, *M* mean, *SD* standard deviation, *HS* high school, *GED* general educational diploma, *SSI* Social Security Income

### Prevalence of cigarette smoking

At baseline, the majority of the participants (84% or 126/150) were current cigarette smokers and 16% (24/150) were non-smokers. Smokers were consuming 8.6 (SD = 7.4) cigarettes per day on average and started smoking when they were 16.6 (SD = 6.4) years old. Their average of duration of smoking was 34.2 (SD = 11.3) years. Of the 24 non-smokers, 66.7% (16/24) were former smokers who started smoking at the age of 17.14 (SD = 5.6) and only 33.3% (8/24) were never smokers.

### Correlates of cigarette smoking

When comparing sociodemographics (Table 1) we found that participants who were current cigarette smokers were more likely to receive supplemental social security income than non-smokers (*p* = .002). No differences were observed in the other sociodemographic characteristics between smokers and non-smokers.

Table 2 summarizes psychiatric and chronic health comorbidities for overall participants and by smoking status. The majority of the participants were diagnosed with a medical condition, with 66.0% any psychiatric condition and 69.3% with any chronic health condition. The most common psychiatric and chronic health conditions were depression (48.7%) and hypertension (38.7%), respectively. Only the percentages of diabetes were significantly different between current and non-smokers at baseline (14.3% vs. 33.3%, *p* = .024).

**Table 2:** Psychiatric and chronic health conditions of the overall study sample and by smoking status

Characteristic	Overall (N = 150) %/M ± SD	Smokers (n = 126) n(%)/M ± SD	Non-smokers (n = 24) n(%)/M ± SD	*p*
Psychiatric conditions				
Depression	73(48.7)	64(50.8)	9(37.5)	.23
Anxiety	42(28.0)	33(26.2)	9(37.5)	.25
Bipolar	22(14.7)	18(14.3)	4(16.7)	.76
Psychosis	7(4.7)	5(4.0)	2(8.3)	.35
Post-traumatic stress disorder	13(8.7)	9(7.1)	4(16.7)	.12
Obsessive-compulsive disorder	1(0.7)	1(0.8)	0(0.0)	.66
Any	99(66.0)	84(66.7)	15(62.5)	.69
QOL - EQ-5D-3L (≥some/moderate problems)				
Mobility	71(47.3)	60(47.6)	11 (45.8)	.87
Self-care	22(14.7)	17(13.5)	5(20.8)	.35
Usual activities	68(45.3)	58(46.0)	10(41.7)	.69
Pain/discomfort	114(76.0)	98(77.8)	16(66.7)	.24
Anxiety/depression	100(66.7)	85(67.5)	15(62.5)	.63
QOL - EQ-5D-3L composite score	0.05(1.29)	0.05(1.35)	0.06(0.9)	.72
BMI	27.8(5.3)	27.7(5.0)	28.4(6.5)	.56
Chronic health conditions				
Arthritis	10(6.6)	8(6.3)	2(8.3)	.71
Asthma/chronic obstructive pulmonary disease	38(25.3)	30(23.8)	8(33.3)	.32
Coronary artery disease	4(2.7)	4(3.2)	0(0.0)	.37
Congestive heart failure	2(1.3)	1(0.8)	1(4.2)	.18
Chronic pain syndrome	9(6)	6(4.8)	3(12.5)	.14
Diabetes	26(17.3)	18(14.3)	8(33.3)	.02
Cirrhosis	41(27.3)	33(26.2)	8(33.3)	.47
Gastro-esophageal reflux disease/peptic ulcer disease	13(8.7)	11(8.7)	2(8.3)	.95
Hyperlipidemia	12(8)	10(7.9)	2(8.3)	.94
Hypertension	58(38.7)	47(37.3)	11(45.8)	.43
Low back pain	23(15.3)	19(15.1)	4(16.7)	.84
Migraine/headaches	5(3.3)	5(4.0)	0(0.0)	.32
Obstructive sleep apnea	1(0.7)	1(0.8)	0(0.0)	.66
Peripheral vascular disease	3(2)	2(1.6)	1(4.2)	.40
Renal insufficiency	6(4)	4(3.2)	2(8.3)	.24
Seizure disorder	5(3.3)	4(3.2)	1(4.1)	.80
Thyroid disease	5(3.3)	5(3.9)	0(0.0)	.32
Any chronic condition	104(69.3)	85(67.4)	19(79.1)	.25

Note: *%* percentage, *M* mean, *SD* standard deviation, *QOL* Quality of life

Table 3 presents results from the urine drug screen and both self-reported current and lifetime alcohol and drug use among participants. Current smokers were more likely to provide a positive drug screen for cocaine (32.5% vs. 12.5%, *p* = .04) and report last 30-day use of cocaine that non-smokers (27.7% vs. 4.1%, *p* = .01) than non-smokers. Rates of alcohol use (34.9% vs. 12.5%, *p* = .02) and drinking alcohol to intoxication (28.5% vs. 0%, *p* = .002) in the past 30 days were significantly higher among cigarette smokers compared to non-smokers. Current smokers also reported a higher number of years drinking to intoxication compared to non-smokers (Mdn = 1.5 vs 0, *p* = .04).

**Table 3:** Current and lifetime substance use of the overall study sample and by smoking status

Characteristic	Overall (N = 150) %/M ± SD/ Md (Q1, Q3)	Smokers (n = 126) n(%)/M ± SD/ Md (Q1, Q3)	Non-smokers (n = 24) n(%)/M ± SD/ Md (Q1, Q3)	*p*
Urine drug screen				
Any drug	74(49.3)	65(51.6)	9(37.5)	.20
Opiates	37(24.7)	32(25.4)	5(20.8)	.63
Cocaine	44(29.5)	41(32.5)	3(12.5)	.04
Benzodiazepines	23(15.3)	19(15.1)	4(16.7)	.76
Amphetamines	17(11.3)	14(11.1)	3(12.5)	.73
Alcohol and drug use				
Past 30 days use				
Alcohol	47(31.3)	44(34.9)	3(12.5)	.02
Alcohol intoxication	36(24.0)	36(28.5)	0(0.0)	.002
Cannabis	44(29.3)	38(30.1)	6(25)	.61
Methadone	148(98.6)	124(98.4)	24(100)	.53
Opiates	33(22.0)	28(22.2)	5(20.8)	.88
Cocaine	36(24.0)	35(27.7)	1(4.1)	.01
Barbiturates	6(4.0)	4(3.1)	2(8.3)	.23
Hallucinogens	5(3.3)	5(3.9)	0(0)	.32
Inhalants	1(0.6)	1(0.7)	0(0)	.66
Lifetime use (years)				
Alcohol	2(0, 7)	2(0, 9.75)	0(0, 3.7)	.11
Alcohol intoxication	1(0, 6.7)	1.5(0 7.0)	0(0, 2)	.04
Cannabis	3.5(0, 15)	4.5(0, 15)	0(0, 22.5)	.80
Methadone	8(3, 12.7)	7(2.25, 15)	10.5(5.25, 23.5)	.08
Opiates	1(0, 5)	1(0, 5)	2(0, 7)	.50
Cocaine	4.5(0, 14.5)	5(0, 15)	2(0, 8.5)	.19
Barbiturates	0(0, 0)	0(0, 0)	0(0, 0)	.07
Hallucinogens	0(0, 1)	0(0, 1)	0(0, 0.25)	.87
Inhalants	0(0, 0)	0(0, 0)	0(0, 0)	.19

Note: *M* mean, *SD* standard deviation, *Md* median, *Q1* quartile 1, *Q3* quartile 3

### HCV treatment outcomes as a function of smoking status

**Fig. 1:**
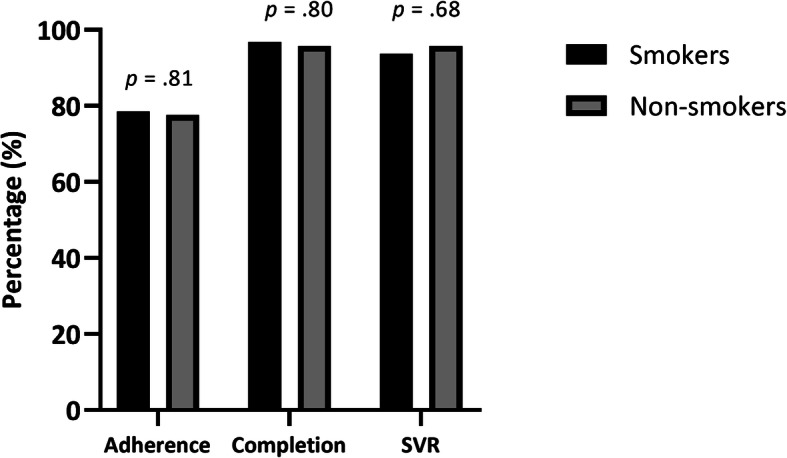
HCV treatment outcomes as a function of smoking status

**Fig. 2:**
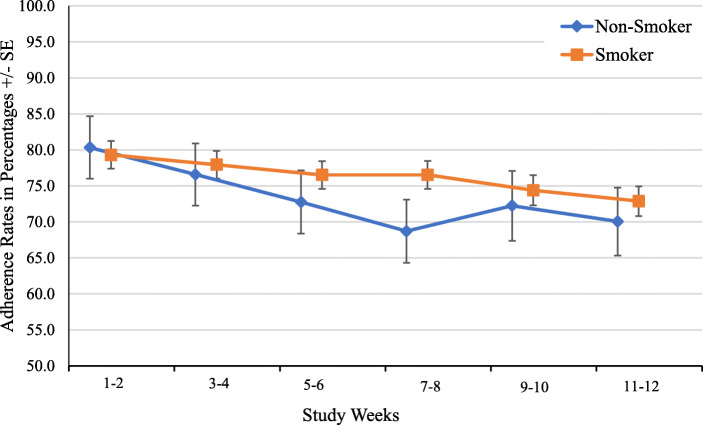
Adherence by smoking status adjusting for study arm, psychiatric conditions, and alcohol intoxication

Figure 1 shows HCV treatment outcomes between smokers and non-smokers. There were no significant differences between smokers and non-smokers in adherence, with percentages being 78.5% (SD = 17.3) and 77.6% (SD = 14.3), respectively (t(145) =0.241, *p* = .81). The adjusted longitudinal trends of adherence were not significantly different between the two groups (*p* = 0.38, Fig. 2). Treatment completion percentages did not differ as a function of smoking status, with 96.8% of the smokers completing treatment compared to 95.8% of the non-smokers (χ^2^ = 0.062, *p* = .80). Finally, SVR rates were not significantly different between smokers and non-smokers, with 93.7% (118/126) of smokers achieving SVR compared to 95.8% (23/24) of the non-smokers (χ^2^ = 0.17, *p* = .68).

### Changes in tobacco use between baseline and 24-week follow-up

Of the 126 smokers who enrolled in the study, 84.9% (107/126) continued smoking an average of 8.6 (SD =7.4) cigarettes a day, 5.6% (7/126) quit smoking cigarettes, and 9.5% (12/126) had unknown smoking status as they were lost at follow-up. Among those who were non-smokers at baseline, 83.3% (20/24) continued not smoking cigarettes and 16.6% (4/24) started smoking an average of 9.7 (SD = 15.5) cigarettes a day. Among the four patients who started smoking at 24-week follow-up, two of them were former smokers and two were never smokers at baseline.

When exploring changes in the percentage of smoking between baseline and the 24-week follow-up, we found that if all 12 participants with missing data at follow-up had continued smoking, the smoking percentages at baseline (84% or 126/150) and the 24-week follow-up (82% or 123/150) would not be statistically significant (McNemar’s test *p* = 0.366).

## Conclusions

This study aimed at examining the prevalence and correlates of cigarette smoking, as well its impact on HCV treatment outcomes among PWID. We note the following five important findings: (1) the majority of the sample were current cigarette smokers; (2) alcohol and cocaine use were associated with cigarette smoking; (3) there was high co-occurrence of both medical and psychiatric conditions; (4) cigarette smoking was not associated with HCV treatment outcomes; and 5) cigarette smoking did not decrease after HCV treatment.

The smoking prevalence found in this study is higher than the estimates reported in earlier studies among HCV-infected persons (i.e., 62.4%) [12]. This finding is likely due to the fact that our entire sample was PWID, a population most severely afflicted with socioeconomic disadvantage. For example, various financial hardships, such as unstable living conditions or unemployment, known to be associated with unsuccessful quit attempts and relapse [29, 30] are common in this sample of HCV-infected PWID. Additionally, this finding may be in part explained because our sample consisted of PWID receiving maintenance treatment for opioid use disorder. Prior research has shown strikingly high prevalence of comorbid cigarette smoking and injection practices [31]. As such, the high co-occurrence in our sample could also be associated with the synergistic effects of smoking and methadone. For example, PWID perceive both behaviors more enjoyable when paired or that smoking reduce methadone aftertaste [32]. Another mechanism that could explain the high prevalence of cigarette smoking among PWID is related to the gateway hypothesis. According to this hypothesis, substance use progresses in sequential stages starting with legal or soft drugs, such as alcohol or tobacco, and later escalating to more addictive illicit drugs later, such as heroin or opioids [33].

Although earlier studies have reported high prevalence of alcohol drinking and cocaine use among HCV-infected PWID [34, 35], we also found that alcohol use and misuse (i.e., heavy drinking) were higher among current cigarette smokers compared to non-smokers among HCV-infected PWID. Several mechanisms may contribute to explain this finding. First, shared, underlying risk factors are a plausible explanation. For example, previous research has demonstrated that negative emotional states, such as stress, are associated with the development and maintenance of drug use and use disorder, including smoking, alcohol use, and cocaine use [36–38]. Second, unique pharmacological interactions between these substances may accelerate their consumption. In this regard, previous experimental studies have robustly shown that consumption of both alcohol and cocaine use increase the rate of cigarette smoking by enhancing the reinforcing effects of nicotine [39, 40]. Third, users of cocaine and alcohol tend not only tend to smoke more, but also frequently consume these substances at the same time [41, 42]. It is possible that the repeated pairing of smoking and both alcohol and cocaine use may result in cigarettes being a stimulus that elicits alcohol and cocaine use behavior and vice versa.

Consistent with prior studies [34, 35], we found high percentages of both psychiatric and chronic health conditions in this sample of PWID. More specifically, we found that 69.3% were diagnosed with a chronic health condition and 66.0% with a psychiatric condition. It should be noted that all of these conditions have been linked to cigarette smoking [43, 44]. It is likely that participants’ smoking may have caused the onset or at least the progression of these conditions. It is also important to acknowledge the high proportion of these participants currently diagnosed with diabetes. Cigarette smoking is a major contributor to adverse liver-related outcomes. Also, there is evidence that the co-occurrence of smoking and HCV may act synergistically [45, 46]. For example, HCV-infected individuals who smoke are at 3 times higher risk to develop hepatocellular carcinoma than those who do not smoke [45]. It is unclear why non-smokers had a higher percentage of diabetes than smokers. It is possible that at least some non-smokers quit because of they were aware of the association of both diabetes and smoking with hepatocellular carcinoma [47].

The finding that neither adherence, treatment completion, nor SVR rates were affected by cigarette smoking merits further comment as well. This result differs from some published studies that have demonstrated that cigarette smoking is associated with lower rates of SVR among HCV-infected individuals [11]. This difference can be explained in part by the several differences in the methodologies applied in these studies. For example, our study ensured the accuracy, consistency, and timeliness of smoking-related data collection as we determined smoking status by asking participants previously validated questions at specific study timepoints. In contrast, earlier studies obtained information about smoking via chart review. Thus, variation in the manner and time which data was gathered as well the discordance about the exact language used to inquire about smoking habits could have impaired the quality of these data. Moreover, these earlier studies were retrospective whereas our study was prospective. Other possible explanation for this result may be related to the models of care of our clinical trial which involved co-located care with or without intensive models of care – group treatment and directly observed therapy. The high rates of cure and adherence found among overall patients in this clinical trial may have undermined our ability to find differences between smokers and non-smokers.

Finally, our finding that HCV treatment was not associated with decrease in cigarette smoking, additional interventions are needed to promote smoking cessation. Since HCV treatment is associated with engagement of OAT patients, evidence-based smoking cessation interventions may be integrated and/or tailored to HCV evaluation, treatment and follow-up visits.

This study has several strengths that should be mentioned. Since this study included a cohort of PWID treated for HCV in OAT settings our findings may inform real-world clinical practice. The longitudinal nature of this study gave us the opportunity to explore changes in cigarette smoking for a 1-year period. This study used multiple sources of data, including blood tests, urine toxicological tests, and questionnaires which allowed us to conduct a comprehensive evaluation of the correlates of cigarette smoking among PWID.

There are some limitations of this study that should be acknowledged. First, cigarette smoking status was only assessed at baseline and follow-up week 24 so it is not possible to explore whether other changes on cigarette smoking occurred between these two points in time. Second, cigarette smoking was determined by relying on self-report and not biochemically verified which may have introduced bias. However, it should be noted that prior studies have demonstrated that self-reported assessment is a valid approach to determine cigarette smoking status [48] and given that our patients were being treated for HCV there is no reason to suspect that these patients would underreport their smoking. Third, combination of never and former smokes, albeit necessary because of small sample sizes, hinders finer classifications that could enable further examination of smoking-related concerns in the HCV-PWID population. Lastly, our findings may not be generalizable to HCV-infected PWID who are not maintained on medications for opioid use disorder (MOUD).

This study has important clinical implications that are worth highlighting. While cigarette smoking did not influence any relevant HCV treatment outcomes in this cohort, we found that those who currently smoke consume both alcohol and cocaine at higher rates. Additionally, we found high percentage of psychiatric and medical conditions in this group of patients. Overall, our findings underscore the importance of addressing cigarette smoking among PWID receiving HCV treatment. DAAs cure almost all HCV-infected patients. However, until other comorbidities are addressed concurrently while HCV treatment is provided, overall morbidity and mortality will continue to rise. Co-localized, multidisciplinary care modes that involve care and treatment of various comorbidities at the same setting, infectious diseases (HIV, HCV), substance use disorders, and medical/psychiatric conditions have been suggested as the most adequate strategy to address health care issues experienced by PWID. The findings of this study highlight that cigarette smoking should also be assessed and addressed while PWID receive HCV care and treatment.

In conclusion, our study found that the majority of this cohort of PWID treated for HCV were current cigarette smokers. We did not find that cigarette smoking was associated with HCV treatment outcomes, although we identified two correlates of cigarette smoking, namely cocaine use and both alcohol use and misuse. HCV treatment was not associated with decreased cigarette smoking. Given the detrimental effects that cigarette smoking and other co-occurring, substance use behaviors (in this study cocaine and alcohol use) have on HCV-infected individuals’ health, it is imperative that clinicians treating HCV also target smoking, especially among PWID.

## Data Availability

The datasets used and/or analyzed during the current study are available from the corresponding author on reasonable request.

## Abbreviations

HCV: Hepatitis C virus
PWID: People Who Inject Drugs
OAT: Opiate Agonist Treatment
SVR: Sustained Virologic Response
